# Characteristics and risk profile of the over fifty adult congenital heart surgical population, a retrospective cohort

**DOI:** 10.3389/fcvm.2025.1568920

**Published:** 2025-06-12

**Authors:** Matthanja Bieze, Oliver Poole, Arshia Delfani, Jane Heggie, Marcus Salvatori

**Affiliations:** ^1^ Department of Anesthesia and Pain Management, Toronto General Hospital and Department of Anesthesiology, and Pain Medicine, Temerty Faculty of Medicine, University of Toronto, Toronto, ON, Canada; ^2^ Department of Anesthesia and Pain Management, QEII Health Sciences Center, Halifax, NS, Canada; ^3^ Department of Cardiac Critical Care, Toronto General Hospital and Department of Anesthesiology, and Pain Medicine, Temerty Faculty of Medicine, University of Toronto, Toronto, ON, Canada

**Keywords:** adult congenital heart disease, perioperative risk, postoperative complications, over 50, cardiac surgery, aging

## Abstract

**Introduction:**

The surgical and medical management of aging patients with adult congenital heart disease (ACHD) continues to innovate to meet the evolving needs of this unique patient population, leading to improved life expectancy and quality of life. However, the ACHD population is characterized by high morbidity and mortality. With this study, we aim to describe patient characteristics and surgical outcomes for the over fifty ACHD cardiac surgical cohort, focusing on risk factors for mortality and major complications.

**Methods:**

This was a retrospective cohort study including ACHD patients undergoing surgical repair from January 2004 to March 2023. Primary outcome was the composite of severe postoperative complications and secondary outcomes were 1-year mortality, ICU stay and hospital length of stay. Descriptive statistics, univariable and multivariable logistic regression models were used.

**Results:**

In the study period, 1381 patients with ACHD underwent cardiac surgery, of which 292 (20.5%) were over 50 years. In the overall group, the most common primary surgery was pulmonary valve replacement in 411 (29.8%), in the over 50 group this was ASD and VSD repairs in 102 (34.9%). The composite of major postoperative complications was different between the overall group and the over 50 years group (10.7% vs. 13.7%; *P* = 0.049), which in the over 50 group was associated with CPB time (180 min vs. 104 min, OR 1.01; 95%CI 1.00–1.03), and preoperative creatinine levels (84 vs. 77, OR 1.01; 95%CI 1.00–1.03). No difference was seen in 1-year mortality (*P* = 0.415).

**Conclusion:**

With careful patient selection and preoperative optimization, surgical risks remain low, even in aging ACHD patients. Although overall mortality rates are low, postoperative complications increase, and patients over 50 with DM, renal failure, long pump runs or postoperative stroke are at highest risk.

## Introduction

The surgical and medical management of congenital heart disease (CHD) continues to innovate to meet the evolving needs of this unique patient population. Surgical refinements improve outcomes while minimizing short- and long-term complications (1). Societal guidelines now inform surgical and medical best practices for almost all CHD (2). Centres of excellence and quaternary referral centres have emerged with specialized care teams focused on the specific needs of both pediatric and adult CHD patients. Large, multicentre databases are used to analyse patient characteristics and outcomes to optimize the surgical approach and timing of intervention. This leads to rapid progress and improvements in patient quality of life. Similarly, routine follow-up and timely re-interventions have extended life expectancy, with 85%–90% of these children living to adulthood (3, 4), commonly past fifty (5).

Even though this trend is encouraging, the ACHD population is characterized by high rates of morbidity and mortality, with multiple readmissions and high healthcare costs (6). These patients come with ACHD-specific comorbidities including congestive hepatopathy, restrictive lung disease, cardiorenal syndrome and vascular abnormalities, which are not adequately reflected in many surgical risk scores. In addition, the growing proportion of ACHD patients reaching later life also develop common age-related comorbidities such as hypertension, diabetes and obesity, which may compound and exaggerate ACHD-related issues.

With this shift within the ACHD population, there is a need for accurate preoperative risk scores to inform the benefit, risk and timing of surgical interventions, especially in the over fifty ACHD population. The current risk scores, including Adult Congenital Heart Surgery (ACHS), Grown-ups with Congenital Heart Disease (GUCH), and PErioperative ACHd (PEACH) scores, lack validation in this specific age group. Toronto General Hospital (TGH) is home to the largest ACHD programs in North America with 4,000 outpatient visits and over 100 ACHD surgeries per year. The total number of ACHD surgeries performed per year at TGH increased over the years from 61 in 2004 to a peak of 112 in 2020, as did the number of surgeries in patients over 50 (17 in 2004 vs. 26 in 2021). With this study, we aim to describe patient characteristics and surgical outcomes for the over fifty ACHD cardiac surgical cohort, focusing on risk factors for mortality and major complications.

## Methods

With institutional ethics approval (CAPCR ID: 15-9178) patients were retrospectively reviewed in three separate databases: TGH Division of Cardiac Surgery, TGH Department of Anesthesia, and the Sickkids Division of Cardiac Surgery. All patients were included at time of surgery in these prospectively maintained registries. All patients older than 17 years of age who underwent ACHD surgery with an open sternotomy or thoracotomy, from January 1st 2004 to the first of April 2023, were included. Patients who underwent multiple procedures were only included for their first case at our Institute, and the primary booking code was used as primary procedure. Of the secondary procedures at the time of the primary procedure, the ablations and coronary artery bypass graft (CABG) were noted. These CABG were performed for coronary artery disease, while the anomalous coronary artery repairs were separately recorded. Patients for chest wall debridement and chest re-explorations for bleeding were excluded, as this was recorded as postoperative complication from the primary surgery. Individual chart review was performed for the following postoperative outcomes: postoperative complications [prolonged ventilation (defined as more than >7 days or 168 h), renal failure requiring dialysis, return to OR for tamponade, stroke, and seizures], 1-year mortality, postoperative length of hospital stay, and length of ICU admission. A composite of postoperative complications (CPC) was used with the following components: renal failure requiring dialysis, prolonged ventilation, in-hospital mortality, stroke, seizures, and return to the OR for tamponade. Additional predictive variables included patient characteristics (BMI, gender, age, comorbidities), surgical indication and procedure, intraoperative data (including aortic cross-clamp and cardiopulmonary bypass times), and perioperative laboratory investigations.

### Statistics

Descriptive statistics were used to give an overview of the baseline characteristics. Results are reported as proportions for categorical variables, and as mean with standard deviation (SD) or median with interquartile range (IQR) for continuous variables. Normally distributed data were compared using a Students-*T*-test, categorical data using the chi-square test or Fishers exact test, and non-normally distributed data using the Mann Whitney U test. Risk factors were considered as independent variables, and their association with CPC was assessed by using a univariable logistic regression analysis. Those variables with a *p*-value <0.1 on univariable logistic regression analysis were analyzed by using multivariable logistic regression analysis to evaluate the variables that independently predict major postoperative complications within 1 month after cardiac surgery.

## Results

### Overall patient group characteristics

Between January 2004 and April 2023, a total of 1,439 cardiac surgery procedures were performed in 1,381 patients with ACHD, of which 292 (20.5%) were over 50 years of age. Over the study period, 48 patients underwent 2 cardiac surgeries at our Institute, and 5 patients underwent 3 procedures. Only the first procedure per patient was used for risk and outcome analysis.

The most common type of primary surgery was pulmonary valve replacement in 411 patients (29.8%); second were the ASD and VSD repairs in 289 patients (20.9%); and third the aortic valve replacement in 194 (13.7%); the full list is available as Supplementary Appendix A. 162 ablations for arrhythmias and 45 CABG were performed at time of the primary procedure.

Median age was 35 (IQR: 26–47), 44.8% were female, and 175 were emergency cases (12.4%). Obesity emerged as a prevalent comorbidity (BMI 25.3; IQR: 22–29.4) and diabetes was present in 52 patients (3.7%). Preserved left ventricular ejection fraction (LVEF) > 55% was seen in 1,074 (75.8%) patients, LVEF 45%–55% in 242 (17.1%), LVEF 30%–44% in 58 (4.1%), and LVEF < 30% in 7 (2.5%) patients. Atrial arrhythmias were known in 302 (21.3%), and median preoperative Hb concentration was 144 g/dl (IQR: 132–154 g/dl). Patient characteristics are summarized in Table 1.

**Table 1:** Patient and surgical characteristics.

Patient characteristics	Overall ACHD group	>50 years ACHD
	*N* = 1,381	*N* = 292
Age in years; median (IQR)	35 (26–47)	56 (53–62)
Gender, female (%)	635 (44.8)	150 (51.4)*
Emergency procedures (%)	175 (12.4)	34 (11.6)
Body Mass Index, median (IQR)	25.3 (22–29)	26.6 (23.6–30.3)
Smoking (%)		
Non-smoker	968 (68.4)	177 (60.6)
Stopped smoking	278 (19.6)	88 (30.1)
Active smoker	135 (9.5)	27 (9.2)
COPD (%)	31 (2.2)	14 (4.8)
Diabetes Mellitus (%)	52 (3.7)	34 (11.6)*
Down syndrome (%)	35 (2.5)	1 (0.3)
Anticoagulation (%)	256 (18.1)	105 (36.0)*
Arrhythmias (%)	302 (21.3)	120 (41.1)*
Ejection Fraction (%)		
>55%	1,074 (75.8)	227 (77.7)
45%–55%	242 (17.1)	54 (18.5)
30–45	58 (4.1)	11 (3.8)
<30%	7 (0.5)	0 (0)
Hemoglobin concentration g/dl, median (IQR)	144 (132–154)	142 (131–152)
Bilirubin, median (IQR)	11 (8–16)	12 (9–18)
Creatinine, median (IQR)	75 (67–87)	77 (69–92)
Cardio Pulmonary Bypass time in min, median (IQR)	109 (72–165)	114 (76–164)
Aortic clamp time time in min, median (IQR)	73 (46–120)	73 (46–119)
Circulatory arrest (%)	59 (4.3)	8 (2.7)
time in min, median (IQR)	18 (7–22)	11 (2–19)

IQR, Inter Quartile Range. Min, minutes.

* *P* < 0.05.

### Outcome after cardiac surgery in the overall patient group

The time on cardiopulmonary bypass (CPB) was 109 min (IQR: 72–165 min), with an aortic clamp time of 73 min (IQR: 46–120 min), circulatory arrest was performed in 59 procedures (18 min; IQR: 7–22 min). The length of stay in the intensive care unit was 1.7 days (IQR: 1–3.8 days), and patients spent 5 h on mechanical ventilation (IQR: 3–13 h), and 7 days in hospital (IQR: 6–13 days). Renal failure requiring dialysis was seen in 44 (3.1%) of patients, stroke in 22 (1.6%), seizures in 7 (0.5%), and return to OR for tamponade in 67 (4.7%), and 1-year mortality was seen in 54 (3.8%). Postoperative results are summarized in Table 2.

**Table 2:** Results.

Postoperative outcomes	Overall ACHD group	>50 years ACHD	*P*-value
	*N* = 1,381	*N* = 292	
ICU in days, median (IQR)	1.7 (1–3.8)	2.0 (1–4)	0.004
Mechanical ventilation Time in hours, median (IQR)	5 (3–13)	6 (4–16)	0.01
Length of hospital stay in days, median (IQR)	7 (6–13)	8 (6–14)	0.96
Dialysis (%)	44 (3.1)	12 (4.1)	0.93
Stroke (%)	22 (1.6)	6 (2.1)	0.73
Seizures (%)	7 (0.5)	1 (0.3)	0.45
Return to OR (%)	67 (4.7)	20 (6.8)	0.35
1-year mortality (%)	54 (3.8)	12 (4.1)	0.415
Composite of major postoperative complications	143 (10.1)	40 (13.7)	0.049

Postoperative outcomes in the overall and the over 50-years group in an unadjusted model.

IQR, interquartile range.

### Patients over 50 years

A total of 292 patients were over 50 years. Similar types of surgeries were seen in this group, with 102 patients undergoing ASD and VSD repairs (34.9%), pulmonary tissue valve replacement in 80 (27.4%), and aortic valve replacement in 27 (9.2%) patients; the full list is available as Supplementary Appendix B. In addition, 48 patients underwent ablation for arrhythmias (16.4% vs. 10.5% in the under-50 group, *P* = 0.004) and 7 CABG (2.4% vs. 3.5% in the under-50 group, *P* = 0.361) at time of the primary procedure. Half of the patient population was female (*n* = 150, 51.4%), 34 procedures were emergency cases (11.6%), BMI was 26.6 (IQR: 23.6–30.3), atrial arrhythmias were seen in 120 patients (41.1%), EF > 55% was seen in 227 (77.7%); EF 45%–55% in 54 (18.5%); and EF 30%–44% in 11 (3.8%) patients, median preoperative Hb was 142 (IQR: 131–152). Patient characteristics are found in Table 1.

### Outcome after cardiac surgery in the over 50 group

The time on cardiopulmonary bypass (CPB) was 114 min (IQR: 76–164 min), with an aortic clamp time of 73 min (IQR: 49–119 min), circulatory arrest was performed in 8 procedures (11 min; IQR: 2–19 min). The length of stay in the intensive care unit was 2.0 days (IQR: 1–4 days), and patients spent 6 h on mechanical ventilation (IQR: 4–16 h), and 8 days in hospital (IQR: 6–14 days). Postoperative dialysis was indicated in 12 (4.1%) of patients and return to OR for tamponade after 20 procedures (6.8%), stroke was seen in 6 (2.1%), seizures in 1 (0.3%), and 1-year mortality in 12 patients (4.1%). Postoperative results are summarized in Table 2. In the unadjusted model the over 50 group was associated with a slight increase in LOS at ICU (1.7 vs. 2.0 days; *P* = 0.004). The composite of major postoperative complications was different between the overall group and the over 50 years group (10.7% vs. 13.7%; *P* = 0.049), although no difference was seen in 1-year mortality (*P* = 0.415).

After adjustment, 1-year mortality was associated with DM (33.3% vs. 10.7%, OR: 5.01; 95%CI 0.75–33.5; although not statistically significant *P* < 0.1), dialysis (50% vs. 2.1%, OR: 31.9; 95%CI 3.04−>50), stroke (16.7% vs. 1.4%, OR: 41.8; 95%CI 3.45−>50). The composite of postoperative major complications was associated with CPB time (180 min vs. 104 min, OR: 1.01; 95%CI 1.00–1.03), and preoperative creatinine levels (84 vs. 77, OR: 1.01; 95%CI 1.00–1.03), also summarized in Table 3.

**Table 3:** Risk factors 1-year mortality and major postoperative complications.

Risk factors	<50 years ACHD *N* = 1,089 (%)		≥50 years ACHD *N* = 292 (%)		*P*-value
	Unadjusted OR (95%CI)	Adjusted OR (95%CI)	Unadjusted OR (95%CI)	Adjusted OR (95%CI)	
1-year mortality					
	*N* = 42 (3.9)		*N* = 12 (4.1)		0.96
Gender *female*	0.97 (0.52–1.8)		0.67 (0.21–2.15)		
Emergency	0.46 (0.22–0.95)*	0.76 (0.28–2.05)	0.58 (0.33–7.39)		
Anticoagulation	4.63 (2.44–8.80)*	2.34 (0.84–6.57)^	5.76 (1.52–21.74)*	2.82 (0.36–22.02)	
Arrhythmias	3.27 (1.71–6.22)*	0.88 (0.31–2.49)	4.54 (1.20–17.14)*	1.6 (0.19–13.89)	
Diabetes	1.48 (0.19–11.36)		4.17 (1.18–14.67)*	5.01 (0.75–33.5)^	
BMI	0.98 (0.93–1.03)		0.92 (0.84–0.999)*	0.96 (0.85–1.09)	
Hemoglobin	1.01 (0.998–1.03)^	0.991 (0.97–1.009)	1.01 (0.98–1.04)		
Creatinine	0.99 (0.98–0.994)*	0.993 (0.98–1.00)^	0.98 (0.96–0.991)*	0.996 (0.97–1.02)	
CPB	0.99 (0.99–0.994)*	0.993 (0.98–0.999)*	0.99 (0.98–0.993)*	0.996 (0.998–1.02)	
X-clamp	0.991 (0.99–0.995)*	1.002 (0.993–1.01)	0.988 (0.98–0.997)*	1.00 (0.98–1.02)	
Renal Failure	34.4 (15.5- > 50)*	15.0 (5.65–39.9)*	45.50 (11.32–182.85)*	31.9 (3.04- > 50)*	
Stroke	9.44 (2.90–30.7)*	1.77 (0.29–10.7)	13.75 (2.25–84.09)*	41.8 (3.45- > 50)*	
Return to OR	7.42 (3.31–16.6)*	4.28 (1.5–12.2)*	2.9 (0.59–14.24)		
Composite major postoperative complications					
	*N* = 103 (9.5)		*N* = 40 (13.7)		0.05
Gender	0.77 (0.51–1.17)		0.94 (0.48–1.83)		
Emergency	2.40 (1.47–3.92)*	1.68 (0.97–2.92)^	2.17 (0.91–5.21)^	1.24 (0.43–3.57)	
Anticoagulation	3.85 (2.45–6.04)*	1.54 (0.83–2.84)	2.49 (1.27–4.89)*	0.74 (0.34–1.61)	
Arrhythmias	2.93 (1.88–4.57)*	1.99 (1.12–3.54)*	1.70 (0.87–3.32)		
Diabetes	1.80 (0.24–13.6)		0.71 (0.27–1.84)		
BMI	0.98 (0.94–1.02)		1.01 (0.95–4.07)		
Hemoglobin	0.994 (0.98–1.004)		0.99 (0.97–1.01)		
Creatinine	1.01 (1.006–1.02)*	1.006 (1.00–1.01)^	1.02 (1.007–1.03)*	1.01 (1.00–1.03)*	
CPB	1.008 (1.005–1.01)*	1.01 (1.008–1.02)*	1.02 (1.01–1.02)*	1.02 (1.008–1.03)*	
X-clamp	1.006 (1.003–1.01)*	0.99 (0.99–1.00)	1.02 (1.01–1.02)*	0.998 (0.99–1.009)	

* Significant with *P* ≤ 0.05.

^ Significance of *P* ≤ 0.1.

### Outcome after cardiac surgery: under 50 years

In the under 50 group a different risk profile for 1-year mortality was seen after adjustment: preoperative creatinine levels [84 vs. 74; OR: 0.993 (95% CI: 0.98–1.00), *P* < 0.05] and longer CPB times (CPB 188 min vs. 107 min, OR: 0.993, 95%CI: 0.98–0.99; *P* = 0.001), as was dialysis [35.7% vs. 1.6%, OR: 15.0; 95%CI (5.65–39.9); *P* < 0.001], and having to return to OR for tamponade (21.4% vs. 3.6%, OR: 4.28; 95%CI: 1.5–12.2; *P* < 0.001). Finally, anticoagulation use was associated after univariable analysis, although it did not reach significance of *P* < 0.05 after adjustment (40.5% vs. 12.8%, OR 2.34; 95%CI 0.84–6.57, *P* < 0.1).

The composite of major postoperative complications after adjustment was associated with arrhythmias (34.0% vs. 14.9%, OR: 1.99; 95%CI: 1.12–3.54); emergency cases (24.3% vs. 11.8%, OR: 1.68; 95%CI: 0.97–2.92, although *P* < 0.1); preoperative creatinine levels (83 vs. 74, OR: 1.006; 95%CI: 1.00–1.01, and finally CPB time (162 min vs. 104 min, OR: 1.01; 95%CI: 1.008–1.02). Summarized in Table 3.

## Discussion

This paper discusses the increasing number of patients who reach beyond 50 years of age with congenital heart disease. It is also one of the first cohorts describing the risk profiles in respect to the overall patient population. In the overall cohort, the most common procedure was pulmonary valve replacement, while the septum defects were most common in the over-50 group. A greater proportion of patients were female in the over 50 group, BMI and LVEF were not different between both groups. However, DM, anticoagulation, arrythmias and ablation procedures were all more common in the over-50 group, as would be expected in the aging population. Patients spend a longer time on the ICU (1.7 vs. 2 days) and on mechanical ventilation (5 vs. 6 h), but this was not deemed clinically relevant. No difference in 1-year mortality was seen, however, the burden of “composite of severe postoperative complications' was higher in the over 50-years patient population [10.1% vs. 13.7%; *P* = 0.049)].

The 1-year mortality was not different between the two groups, however, the risks leading up-to this 1-year mortality did differ. In the younger group, patients had a higher risk of mortality when on anticoagulation, when on dialysis and when they had to return to the OR for tamponade. Patients over 50-years were at higher risk for 1-year mortality with DM, dialysis and after stroke. When patients reach an older age, their comorbidities will most likely contribute to their risk of mortality. The likelihood of multiple cardiac procedures increases as patients get older, as for example MAZE for AF was more common as atrial dilatation develops over time in this post-repair or structurally challenged population. These risks are greater in the ACHD group compared to the general population, with much higher risks of developing ischemic stroke in the <65 years group, which is estimated to only increase in the years thereafter (7). The incidence of stroke in the overall ACHD group with AF was 3.11 per 1,000 (one in 30 patients under the age of 47), leading to an adjusted HR of 5.16 (95% CI: 1.52–17.46). These numbers would increase further with the more complex congenital lesions. When compared to the control group with AF, the incidence of ischemic stroke was 0.73 per 1,000 patients; this is a considerable five-time increased risk which calls for ongoing follow-up and specialized care. In our cohort 1.6% of patients had a stroke after surgical repair, however, this was not in just the ACHD with AF, but in the overall ACHD group.

Additional risks for mortality were described by Afilalo et al. (8) who showed dementia, gastrointestinal bleeds and chronic kidney disease to be strong predictors of mortality in this aging population. Coronary artery disease is another age-related and growing concern (9, 10). In our cohort there was no statistical difference in patients undergoing CABG before or after the age of fifty. However, this number might be underestimated as patients may undergo CABG by non-congenital surgeons or at other cardiac centers, as this often does not involve congenital expertise. These numbers also indicate that a greater proportion of ACHD patients undergo CABG procedures at a young age (11). This increased risk is like most things multifactorial: these patients have a higher risk of developing atherosclerosis as their anatomy often differs, or is changed, with significant postsurgical injury, and abnormal (bi)ventricular function. In addition, there can be increased inflammation and cytokine levels, which play a role in the development of coronary artery disease over time, now also supported and addressed in the consensus by the European Society of Cardiology (10–13).

Limitations of the study are the following: the retrospective nature of the study, which inevitably led to missing data and inability to assess for additional outcomes. It would have been beneficial to be able to evaluate the degree of RV dysfunction as a high percentage of the patients had disease effecting the right heart, with the greatest indication for surgery being PVR. Similarly, although we sought to compare sequelae of RV dysfunction such as congestive hepatopathy, pre-operative liver enzymes were inconsistently collected which often precluded MELD score calculations. Finally, although we had hoped to compare our outcomes to those predicted by risk prediction models, we could not run the Euroscore as not all required inputs were recorded in our database.

There is a great overall improved safety with low risks to cardiac surgery even in the ACHD population (14) Excellent patient selection and preoperative optimization have contributed to that fact. From a large recent study we know that 75% or more of patients with ACHD whom are alive at the age of 18 will live into their sixties (15). We also know these older patients are more satisfied in life, with less anxiety than their younger counterparts, even if they are limited by physical hardship (16). Quality of life after cardiac surgery in ACHD shows improved patient-reported outcomes across mental, physical, and social domains that lasts the first postoperative year (17). However, in the words of Mandalenakis et al. we should consider the operated patients “treated”, but not “cured” (18) and specialized follow-up remains essential to maintain this level of excellence.

## Conclusion

In conclusion, this retrospective study provides valuable insights into the safety and effectiveness of cardiac surgery in the over fifty ACHD population. The findings underscore that with careful patient selection and preoperative optimization, surgical risks remain low, even in this complex cohort. Although over morality rates are low, patients over 50 with DM, dialysis-dependence or postoperative stroke are at highest risk and require particular attention. Future research should aim to validate risk prediction models for the over fifty ACHD population, to further refine treatment strategies.

## Data Availability

The datasets presented in this article are not readily available due to patient privacy, ethical regulations and strict regulations of the University. Requests to access the datasets should be directed to the corresponding author.
