# Correction: Characteristics and risk profile of the over fifty adult congenital heart surgical population, a retrospective cohort

**DOI:** 10.3389/fcvm.2025.1658416

**Published:** 2025-08-07

**Authors:** Matthanja Bieze, Oliver Poole, Arshia Delfani, Jane Heggie, Marcus Salvatori

**Affiliations:** ^1^ Department of Anesthesia and Pain Management, Toronto General Hospital and Department of Anesthesiology, and Pain Medicine, Temerty Faculty of Medicine, University of Toronto, Toronto, ON, Canada; ^2^ Department of Anesthesia and Pain Management, QEII Health Sciences Center, Halifax, NS, Canada; ^3^ Department of Cardiac Critical Care, Toronto General Hospital and Department of Anesthesiology, and Pain Medicine, Temerty Faculty of Medicine, University of Toronto, Toronto, ON, Canada

**Keywords:** adult congenital heart disease, perioperative risk, postoperative complications, over 50, cardiac surgery, aging

Affiliation 2 for author Oliver Poole was incorrect. The correct affiliation 2 is “Department of Anesthesia and Pain Management, QEII Health Sciences Center, Halifax, NS, Canada”.

The original version of this article has been updated.

