# Supplementary material for: Characteristics and risk profile of the over fifty adult congenital heart surgical population, a retrospective cohort
Source: Front Cardiovasc Med. 2025 Jun 12;12:1568920. doi: 10.3389/fcvm.2025.1568920 (PMC12198246; doi:10.3389/fcvm.2025.1568920)
Supplement: Supplementary file 1 [file Datasheet1.pdf]

Supplementary Material 1

Type of surgery Overall ACHD group

	Frequency	Percent %
PAPVD-ASD repair	111	7.8
ASD-primum repair (partial AVSD)	53	3.7
ASD-secundum repair	50	3.5
ASD-sinus venosus repair	24	1.7
AVSD-complete repair	9	.6
VSD-perimembranous repair	40	2.8
VSD-outlet repair	2	.1
PAPVD-isolated repair	23	1.6
Aortic valve replacement (mechanical)	130	9.2
Mitral valve replacement (mechanical)	32	2.3
Tricuspid replacement (tissue)	36	2.5
Tricuspid replacement (mechanical)	15	1.1
Tricuspid annuloplasty	3	.2
Pulmonary valve replacement (tissue)	411	29.0
Mitral valvuloplasty	16	1.1
Aortic valvuloplasty	7	.5
Aortic valve replacement (tissue)	64	4.5
Cavopulmonary anastomosis	2	.1
Coronary repair ALCAPA(anomalous origin from PA)	7	.5
Coarctation repair-bypass or tubular graft	13	.9
Conduit RV-PA - valved	65	4.6
Pulmonary artery banding-MPA	7	.5
Unifocalization of PA	2	.1
Tetralogy repair	15	1.1
Fontan conversion	21	1.5
Patch aortoplasty	5	.4
Coarctation repair-end to end	1	.1
Ascending aortic replacement	19	1.3
Ablation of arrhythmia	2	.1
Unroofed Coronary Sinus repair	2	.1
LVOT fibromyectomy	38	2.7
RVOT-resection (indirect)	16	1.1
Conduit LV-PA - valved	5	.4
Coronary artery repair	16	1.1
Pulmonary arterioplasty	6	.4
Tricuspid valvuloplasty	22	1.6
Aortic arch repair	4	.3
Aneurysm repair-sinus of Valsalva	5	.4
Central Shunt with graft	1	.1
Mitral valve replacement (tissue)	4	.3

TGA repair-double switch	1	.1
Heart transplant	25	1.8
Pulmonary valvuloplasty	1	.1
Vascular ring repair	9	.6
Pacemaker-primary implant	2	.1
Pulmonary valve replacement (mechanical)	2	.1
Pulmonary embolectomy	1	.1
Aneurysm repair-aortic	7	.5
Baffle revision	2	.1
Mitral annuloplasty	3	.2
Pericardectomy	2	.1
Tumor excision	1	.1
Ventricular assist device-VAD	3	.2
Cor triatriatum repair	1	.1
Subaortic septal patch (Konno)	2	.1
Thrombectomy	1	.1
ROSS procedure	2	.1
TAPVD repair	1	.1
TGA repair-Mustard operation	1	.1
Bentall procedure with mechanical vavle, replacment of RV-PA conduit, sp Ross procedure	2	.1
Other	8	.6
Total	1381	97.5

Supplementary Material 2

Type of surgery >50 years ACHD group

	Frequency	Percent %
PAPVD-ASD repair	48	16.4
ASD-primum repair (partial AVSD)	17	5.8
ASD-secundum repair	13	4.5
ASD-sinus venosus repair	10	3.4
AVSD-complete repair	2	.7
VSD-perimembranous repair	12	4.1
PAPVD-isolated repair	3	1.0
Aortic valve replacement (mechanical)	15	5.1
Mitral valve replacement (mechanical)	10	3.4
Tricuspid replacement (tissue)	13	4.5
Tricuspid replacement (mechanical)	3	1.0
Tricuspid annuloplasty	1	.3
Pulmonary valve replacement (tissue)	80	27.4
Mitral valvuloplasty	4	1.4
Aortic valvuloplasty	1	.3
Aortic valve replacement (tissue)	12	4.1
Cavopulmonary anastomosis	1	.3
Coronary repair ALCAPA(anomalous origin from PA)	1	.3
Coarctation repair-bypass or tubular graft	2	.7
Conduit RV-PA - valved	3	1.0
Tetralogy repair	3	1.0
Fontan conversion	1	.3
Ascending aortic replacement	3	1.0
LVOT fibromyectomy	8	2.7
RVOT-resection (indirect)	3	1.0
Coronary artery repair	2	.7
Pulmonary arterioplasty	1	.3
Tricuspid valvuloplasty	6	2.1
Heart transplant	6	2.1
Vascular ring repair	1	.3
Pulmonary valve replacement (mechanical)	1	.3
Aneurysm repair-aortic	1	.3
Thrombectomy	1	.3
TAPVD repair	1	.3
Bentall procedure with mechanical vavle, replacment of RV-PA conduit, sp Ross procedure	1	.3
Other	2	.7

	Total	292	100.0
